# Editorial: Evolutionary computation-based machine learning and its applications for multi-robot systems

**DOI:** 10.3389/fnbot.2023.1177909

**Published:** 2023-04-24

**Authors:** Lianbo Ma

**Affiliations:** Software College, Northeastern University, Shenyang, China

**Keywords:** evolutionary computation, machine learning, multi-robot systems, optimization, control system

With the explosive of communication technology, the optimization and application of multi-robot systems becomes a challenging issue. From an algorithm design perspective, evolutionary computation-based machine learning (ECML) methods show advantages over classical integer programming, Markov decision making, and auction algorithms in dealing with complex optimization problems, with the most prominent advantage being that ECML has a strong search capability that can largely reduce the computational cost of cluster node analysis. Therefore, this issue aims to provide a platform for the ECML methods and their application in multi-robot systems, demonstrating the possibilities of ECML as much as possible.

Chen et al. design a quantum particle swarm optimization (QPSO)-based model predictive control (MPC) tracking algorithm for cable-driven continuum robots (CDCR) to improve control stability and trajectory tracking accuracy under constraints. The researchers analyze the positive reflections between actuation space, joint space and working space using the segmented constant curvature method and the chi-square coordinate transformation for more accurate kinematic analysis of the CDCR. QPSO is employed in the rolling optimization of MPC because of its global optimization performance, robustness, and fast convergence and thus improves the performance of MPC in complex tracking tasks. After simulation and operational experiments, it is verified that the designed QPSO-MPC can meet the requirements of higher control stability and higher trajectory tracking accuracy compared with MPC and particle swarm optimization (PSO) based MPC.

Li et al. focus on unmanned aerial vehicles (UAVs) to provide emergency rescue services for the affected people. For the problem of scattered distribution of people in the affected areas, the researchers propose a global optimal target poi clustering model based on particle swarm optimization (PSO) to centralize the population. In addition, a green energy consumption calculation model proposed in the paper considers the distance between each UAV and the target POI, the power of each UAV, and the priority of the target POI, and selects the appropriate UAVs to perform missions to the target POI to ensure that all UAVs have low energy consumption when completing their missions. Finally, the researchers design the cross-UTPA multi-drone collaborative path planning algorithm, which has no action space limitation for policy learning and can be multi-tasked in parallel, improving the efficiency and generalization of sample processing. Validation has shown that the study improves the success rate of the UAV in reaching the target location and reduces the energy requirements of the UAV, thus improving rescue efficiency.

Jia et al. proposed a deep learning neural model called the Vital Information Matching Feedback Self-Tuning Network (VIM-Net) to overcome the problem of merely comprehensive feedback of intrinsic information through simple data augmentation or extension. VIM-Net consists of two main matching feedback modules, a visual matching feedback module (V-mat) and a trajectory matching feedback module (T-mat), where the former is responsible for matching the visually recognized target information with the entity information extracted by the command, and the latter is responsible for matching the serialized trajectory features with the direction of motion of the command. In an attempt to measure model better, the researchers select Matterport3D simulator and perform several ablation experiments and comparative experiments based on Room-to-Room (R2R) benchmark dataset, recording the final navigation effect information. The results of this investigation suggest that VIM-Net is effective on the task.

Wan et al. provide a new approach to designing patterns that is conductive on multi-modal tasks. In order to improve the accuracy and speed of generation, the method chooses Max Pooling theory and discrete Laplacian differential operators as a means to compress and enhance the target background. The background pixel primitives are treated as objects before the sample data is computed. After considering the artifactual pattern and background, the researchers chose the K-means clustering principle to perform and iterate over the computed data, and evaluated the model using color similarity and shape similarity. The details in the results show that the new generation method works well and provides important insights into pattern generation.

To sum up, four articles have been published on this Research Topic, including the findings of scholars and industry personnel in the relevant fields. These findings help readers to better understand and learn the latest knowledge in the related fields and thus stimulate more new discoveries.

## Author contributions

The author confirms being the sole contributor of this work and has approved it for publication.

